# Analysis of enamel development using murine model systems: approaches and limitations

**DOI:** 10.3389/fphys.2014.00313

**Published:** 2014-09-17

**Authors:** Megan K. Pugach, Carolyn W. Gibson

**Affiliations:** ^1^Department of Mineralized Tissue Biology, The Forsyth Institute, Harvard School of Dental Medicine, Harvard UniversityCambridge, MA, USA; ^2^Department of Anatomy and Cell Biology, School of Dental Medicine, University of PennsylvaniaPhiladelphia, PA, USA

**Keywords:** enamel development, transgenic, knockout, knockin, amelogenin, mineralization

## Abstract

A primary goal of enamel research is to understand and potentially treat or prevent enamel defects related to amelogenesis imperfecta (AI). Rodents are ideal models to assist our understanding of how enamel is formed because they are easily genetically modified, and their continuously erupting incisors display all stages of enamel development and mineralization. While numerous methods have been developed to generate and analyze genetically modified rodent enamel, it is crucial to understand the limitations and challenges associated with these methods in order to draw appropriate conclusions that can be applied translationally, to AI patient care. We have highlighted methods involved in generating and analyzing rodent enamel and potential approaches to overcoming limitations of these methods: (1) generating transgenic, knockout, and knockin mouse models, and (2) analyzing rodent enamel mineral density and functional properties (structure and mechanics) of mature enamel. There is a need for a standardized workflow to analyze enamel phenotypes in rodent models so that investigators can compare data from different studies. These methods include analyses of gene and protein expression, developing enamel histology, enamel pigment, degree of mineralization, enamel structure, and mechanical properties. Standardization of these methods with regard to stage of enamel development and sample preparation is crucial, and ideally investigators can use correlative and complementary techniques with the understanding that developing mouse enamel is dynamic and complex.

## Introduction

Because murine teeth have significant similarities to those of humans, murine models have been generated to study effects of deleting or altering specific protein coding genes, followed by detailed phenotypic evaluation. Mice are ideal for this purpose as their genetics have been well characterized and gene transfer technology is highly developed. The focus will be on genes encoding enamel proteins which have significant homology to those of humans, including secreted proteins amelogenin, ameloblastin, enamelin, proteases MMP20 (matrix metalloproteinase 20) and KLK4 (kallikrein 4) and cell-associated proteins ODAM (odontogenic ameloblast-associated protein) and AMTN (amelotin) (see Table 1 for list of abbreviations) (Hu et al., 2005; Wright et al., 2009; Holcroft and Ganss, 2011; Dos Santos Neves et al., 2012; Bartlett, 2013).

**Table 1:** **List of Abbreviations**.

MMP20	Matrix metalloproteinase-20
KLK4	Kallikrein-related petidase 4
ODAM	Odontogenic ameloblast-associated protein
AMTN	Amelotin
AMELX	Amelogenin
M180	Murine 180 amino acid amelogenin
LRAP	Leucine rich amelogenin peptide
TRAP	Tyrosine rich amelogenin peptide
M180Δ A-FLAG	Amelogenin with engineered N-terminal changes and reporter
M180Δ B-FLAG	Amelogenin with engineered C-terminal changes and reporter
CTRNC	Transgene with the C-terminus of amelogenin (M180) deleted
DSPP	Dentin sialophosphoprotein
FAM20C	Family with sequence similarity 20 gene
ENAM	Enamelin
AMBN	Ameloblastin
KO	Knockout or null mutation, designated -/- (+/- is heterozygous gene)
WT	Wild-type
PCR	Polymerase chain reaction
Cre-Lox	System to generate site specific recombinations in DNA
K14	Keratin-14 gene promoter
LacZ	Gene encoding beta-galactosidase
E18.5	Mouse at embryonic age day 18.5
AI	Amelogenesis imperfecta
HA	Hydroxyapatite
Tg	Transgene
SEM	Scanning electron microscopy
BSE	Backscattered SEM
FESEM	Field-emission SEM
TEM	Transmission electron microscopy
μCT	Micro-computed tomography
VOI	Volume-of-interest
RGB	Red, green and blue
WIC	Whiteness index
CIE	International Commission on Illumination
LAB	Color space where L is lightness, a is red/green and b is yellow/blue
ICC	Immunocytochemistry

Enamel phenotypes from rodents with genetically modified enamel genes have been analyzed using a wide range of methods to report and compare the physical properties of enamel such as mineral density, structure, mechanical integrity, and color. Mineral density of mouse enamel has been measured by micro-computed tomography (μCT) (Schmitz et al., 2014), backscattered scanning electron microscopy (BSE) (Smith et al., 2011b), and ashing (heating) (Smith et al., 2011a). Enamel structure has been analyzed by a variety of microscopy techniques and magnifications, but the most appropriate microscopy and magnification depends heavily on the structural information (i.e., enamel rods, enamel crystallites, enamel proteins) and stage of enamel development of interest to the investigator. Enamel mechanical integrity from genetically modified mice has been measured by microhardness (Sharma et al., 2011; Lacruz et al., 2012; Kweon et al., 2013) and nanoindentation (Fong et al., 2003; Li et al., 2008; Pugach et al., 2013). It is critical to understand the limitations of these techniques so that they can be utilized correlatively to complement each other, and so investigators can share and compare data from different rodent models, in an effort to more completely understand enamel formation and mineralization.

## Generation of mice with genetically modified enamel genes

### Transgenic mice

A transgene can be generated on a plasmid in such a way that the regulatory region that directs tissue specificity is inserted upstream of a protein coding region for expression in that tissue. This plasmid is injected into fertilized mouse eggs and then the eggs are re-implanted into a foster mother mouse (Doyle et al., 2012). This DNA will insert randomly into the genome so that it must contain sufficient regulatory material to direct expression without major influence by the site of insertion. The DNA can insert in multiple copies as well as in multiple locations in the genome, and frequently higher copy number leads to higher expression of the transgenic protein. However, if the DNA inserts into a required gene, a secondary phenotype may be observed unrelated to the transgenic protein. For this reason, at least three independent transgenic pups are generally analyzed to avoid problems related to site of insertion. An advantage is that this approach may allow low, medium, and high levels of transgene expression to be studied, but in the presence of expression by the endogenous gene.

A simple model system involves a transgenic mouse that expresses a detectable reporter protein to indicate where a specific gene regulatory sequence is active, such as was done for the amelogenin gene promoter (Chen et al., 1994). Transgenic reporters have included β-galactosidase, luciferase, human growth hormone, and thymidine kinase (Al-Shawi et al., 1988; Dilella et al., 1988; Sweetser et al., 1988; Theopold and Kohler, 1990).

Another common strategy is to generate a transgenic mouse that overexpresses a normal protein in the endogenous tissue such as amelogenin, LRAP or TRAP (small amelogenins), ameloblastin, enamelin, MMP20, amelotin, DSPP (Paine et al., 2003, 2004, 2005; Gibson et al., 2007; Lacruz et al., 2012; Stahl et al., 2013; Hu et al., 2014; Shin et al., 2014). It is also possible to express a transgene more broadly so that the transgenic protein is expressed in a tissue different from or in addition to the endogenous, e.g., by use of the K14 promoter (Atsawasuwan et al., 2013). Overexpression of a transgene with a mutation or deletion to interfere with the function of the endogenous protein in a dominant negative strategy has been used followed by phenotypic analysis in order to better understand protein function (Dunglas et al., 2002; Gibson et al., 2007; Pugach et al., 2010; Chen et al., 2011).

Transgenes are relatively unlikely to cause a lethal event, but lack of detectable transgenic expression, or expression in unexpected tissues are both common. These observations related to expression can be confusing but may lead to new ideas concerning where the gene is normally expressed, additional functions during development, or gene motif requirements for accurate tissue specific and level of expression. Transgene overexpression may not however accurately reflect the human condition.

#### Transgene expression levels

A problem in use of transgenic mice is difficulty in replicating the level of expression of the endogenous gene. In the case of amelogenins, since there is one primary RNA transcript but many amelogenin mRNAs, the appropriate expression level for an individual protein is cause for debate as it is not clear whether matching the endogenous level is possible. In rescue experiments, the high expression level of one of the amelogenin transgenes can rescue the phenotype when mated with amelogenin null mice, but the low expressor is unable to rescue significantly. Various levels of transgene expression have allowed determination of the amount of protein that either damages the normal phenotype or rescues the null phenotype (Li et al., 2008; Chun et al., 2010; Shin et al., 2014).

### Knockout mouse models

A “knockout” is a term for a mouse with a single mutated gene so that no protein is expressed, also referred to as a “null” mutation (Mansour et al., 1988). A null mutation can provide relatively straight forward means to understand consequences of lack of a particular protein. If the result is a lethal event, this approach can still provide information on when protein function begins to be important, and tissues impacted by lack of the protein. Loss of a structural protein in a mineralized tissue may lead to an obvious defect, or to a defect that becomes apparent in the presence of a stressor. Mutations in enamel proteins frequently lead to enamel that is chalky in appearance and subject to attrition. This knockout approach can be designed to duplicate human pathology by deletion of a structural protein or enzyme predicted to be important.

In the simplest situation, a plasmid vector that includes antibiotic selection cassettes is generated to contain a segment of the gene of interest with an engineered mutation such as a deletion or stop codon to prevent expression. This plasmid is introduced into cultured embryonic stem cells followed by chemical or antibiotic selection *in vitro*. Clones that grow are tested to determine whether the engineered gene has replaced the endogenous gene. Clones that seem perfect by Southern blot and PCR of genomic DNA are transferred into blastocysts and the recombinant structure is implanted into a surrogate female mouse (Doyle et al., 2012). The pups that are born are tested by PCR using DNA isolated from tail tissue to determine the presence of the mutation in individual pups. Positive mice are mated with wild-type mice to ascertain that the mutation can be passed on to offspring, and has been inserted correctly into the mouse genome. Individual strains can be developed from positive mice; usually several mouse strains are evaluated phenotypically for effects of the deleted gene.

Several problems can be encountered using this approach. The amelogenin null mouse was generated by deletion of parts of exon 2 and intron 2, and exon 2 containing the start sequence in present in all cDNAs sequenced to date (Gibson et al., 2001). This deletion allowed amelogenin RNA to be produced that lacked exon 2, but was not translated.

The ameloblastin KO originally reported (Fukumoto et al., 2004) was actually a partial deletion leading to expression of truncated protein (Wazen et al., 2009). A different kind of problem is associated with the MMP20 KO as the KO enamel delaminates from dentin so that it is difficult to study the enamel structure (Caterina et al., 2002).

### Rescue experiments

In addition to direct analysis, transgenic and null mice can be mated together to perform phenotypic rescue experiments. An example is rescue of the amelogenin null hypoplastic phenotype by single amelogenin proteins. Although there are more than 15 amelogenin mRNAs, this rescue required only one or two transgenes for significant improvement of the enamel layer (Li et al., 2008; Gibson et al., 2011). As mentioned above, transgene expression level is also critical for efficient rescue for ameloblastin, enamelin, and MMP20 null mice (Chun et al., 2010; Hu et al., 2014; Shin et al., 2014).

### Secondary phenotypes from whole-body gene deletion

The above strategy is useful when ablation of a protein does not constitute a lethal event. In that case, other strategies such as Cre-lox mediated tissue specific deletions can be attempted. Unexpected phenotypes may develop in a tissue that was not previously known to express the deleted gene.

**Figure 1:**
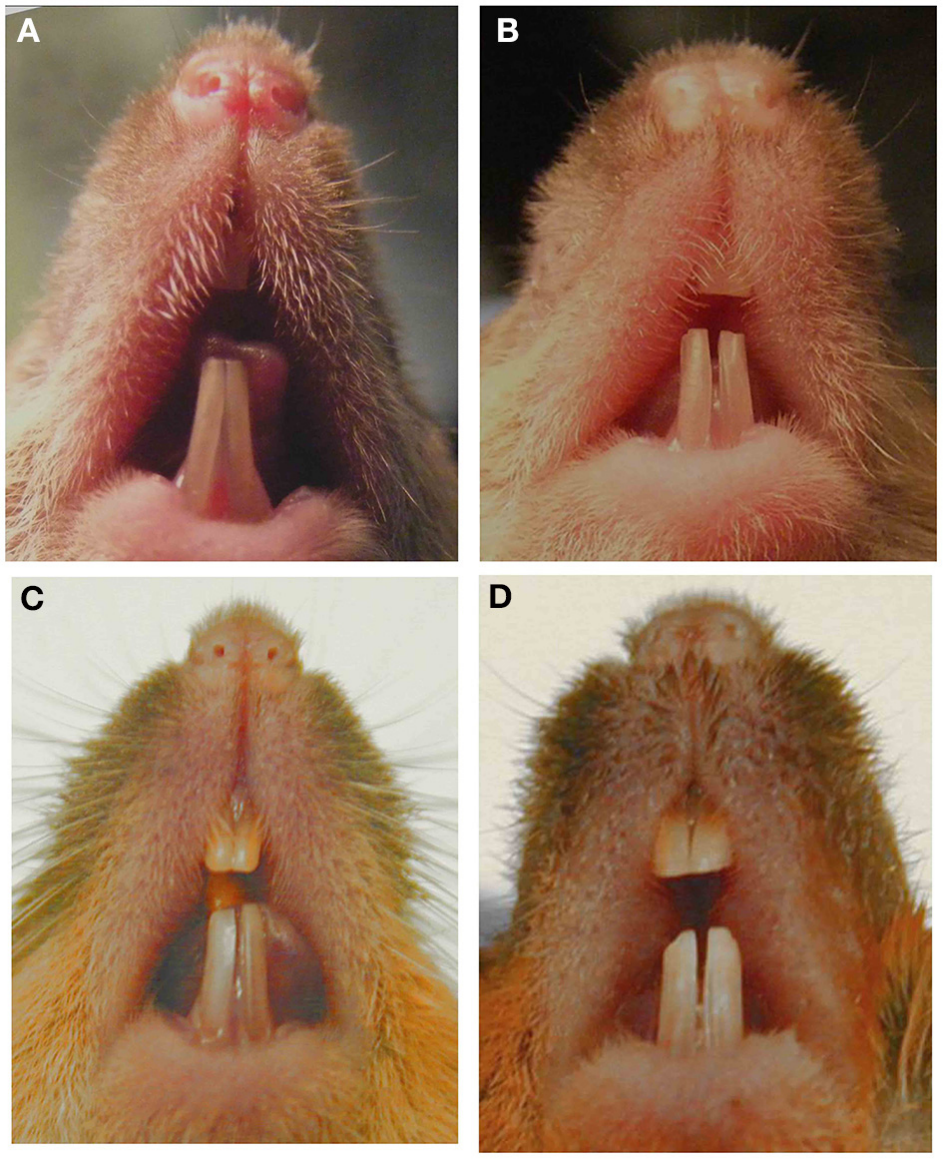
**Images of murine incisors from Amelx null mice**. Null mice with mixed genetic background and phenotypic variability in **(A)** 7-week male, **(B)** 8-week male, **(C)** 6-month male, **(D)** 6-month female. This figure was originally published in Li et al. (2014).

An unexpected phenotype can also be observed when a null mutation is moved to a mouse with a different genetic background. Most null mutations are generated in mice with mixed background but then are transferred by repeated mating and screening to C57Bl/6 or another inbred strain in order to reduce phenotypic heterogeneity. Many examples have been reported where inbred mice have a phenotype different from that in the original mixed background strain. Phenotypic heterogeneity of the incisors can be easily observed within a group of adult amelogenin null mice with mixed genetic backgrounds (Figure 1). For example, when MMP20 or amelogenin null mutations were moved to a different genetic background, the enamel phenotype was altered (Li et al., 2014; Bartlett, personal communication).

The above limitations could be seen as supplying new knowledge difficult to obtain by other means, such as *in vitro* experiments. However, an important consideration is the expense of maintaining colonies of mice, especially those having small or infrequent litters, or those with lethal outcomes. When mating efficiency is reduced in null mice, they can be maintained as heterozygotes (+/−) and then mated together to produce +/+, +/−, and −/− offspring, that is the controls are generated within the litter.

### Cre-lox tissue specific deletions

To generate a tissue specific targeted gene deletion using the Cre-lox system, two mice are required (Doyle et al., 2012). One mouse will have a transgene that expresses the Cre-recombinase under control of a tissue specific promoter. That mouse is mated to a mouse with LOX-P sites inserted within the gene of interest in such a way that deletion of the gene segment between LOX-P sites will lead to a tissue specific null mutation in the offspring that have both Cre and LOX-P genes. This strategy may avoid lethality as the Cre recombinase, under control of a tissue specific promoter, may be expressed later in development and in only the target tissues.

Using the Cre-lox approach, a deletion was generated in the ARHGAP6 gene which also removed the amelogenin gene localized to an ARHGAP6 intron, leading to an enamel defect (Prakash et al., 2005). A mouse that expressed the Cre recombinase under control of the Amelogenin regulatory sequences was mated with mice with a floxed TGFβ receptor II gene to generate enamel pathology due to deletion of receptor activity (Cho et al., 2013). Mice with the K14 promoter regulated Cre recombinase were mated to floxed Rac1 mice leading to ameloblast cell changes and enamel defects (Huang et al., 2011). K14-Cre was also used to delete FAM20C, again leading to enamel defects (Wang et al., 2013).

### Knock-in approaches

This strategy is similar to that used for a knock-out mouse, except the vector does not contain a deletion to generate a null mouse. Instead the knock-in vector replaces the endogenous gene with a gene segment with a mutation in a region of interest of the translated protein or with a reporter gene. This mouse will express a mutated protein or reporter in place of the wild-type protein.

N- or C-terminal coding regions of the amelogenin gene were removed in a knock-in model that addressed function of domains of the amelogenin protein (Zhu et al., 2006). The enamelin gene was replaced by the LacZ gene to generate a knock-in mouse with enamel defects (Hu et al., 2008). A similar approach was used for a knock-in of the KLK4 gene (Simmer et al., 2009). This approach allows detection of tissue specific gene expression while generating a null mutation in the gene of interest.

## Analysis of genetically modified rodent enamel

### Mineral content

The mineral content of wild-type rodent enamel has been reported to range from 86.2% (by volume) (Angmar et al., 1963) to 95.06% (by volume) (Schmitz et al., 2014), values that depend greatly on the enamel composition model used. Rodent enamel has a very broad range of mineral content, both during development (molars) and in continuously erupting incisors. When enamel is affected by genetically altering enamel genes in rodents, mineralization defects are common. However, assessment of degree of mineralization in poorly mineralized enamel is technically challenging. Hydroxyapatite (HA) content in enamel can be quantified through direct and indirect methods.

**Figure 2:**
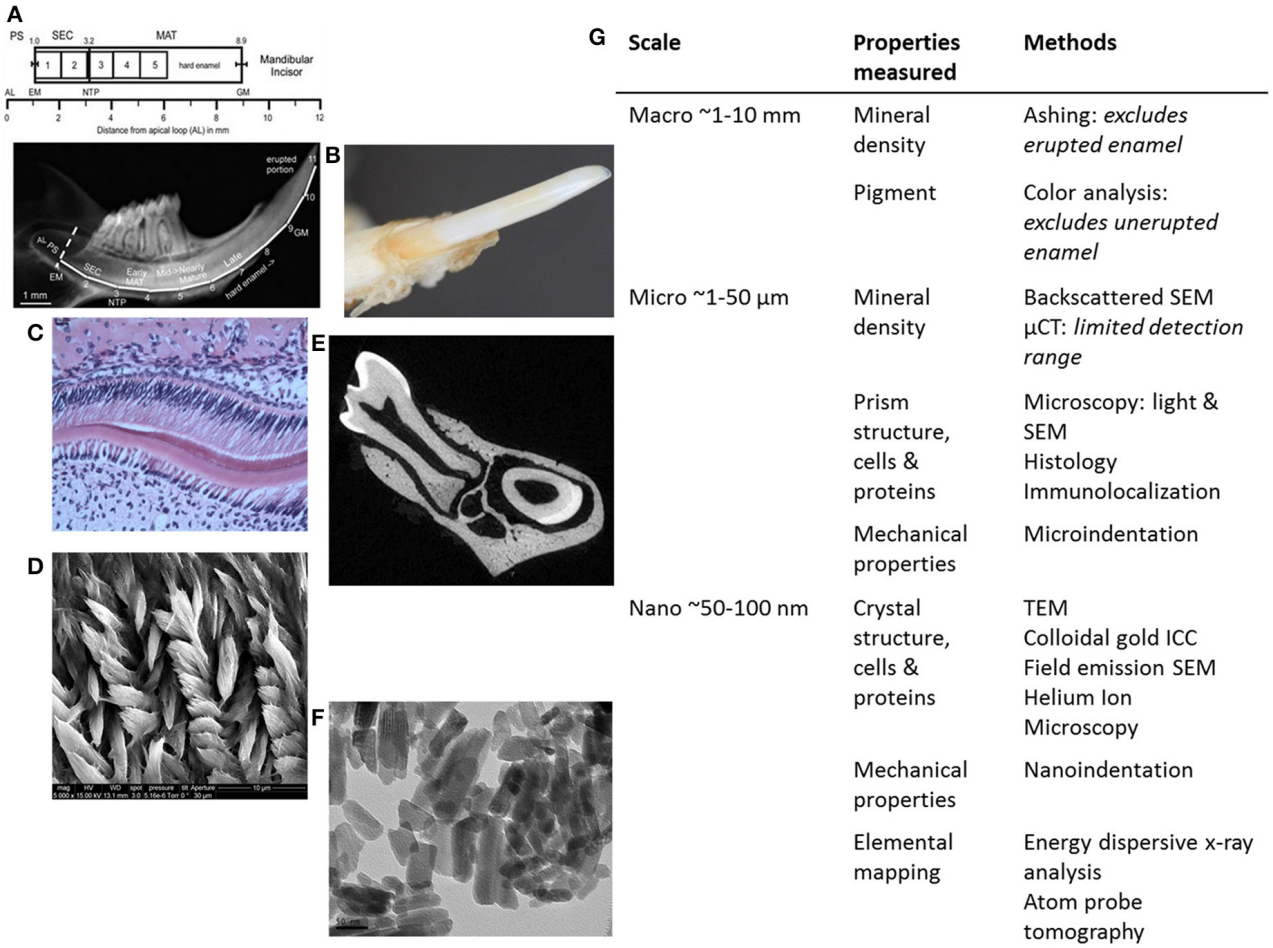
**Summary of methods for analyzing genetically modified rodent enamel. (A–F)** Representative images of WT mouse enamel analyzed by different methods are shown, with the corresponding table **(G)** in which methods are separated according to scale (mm, μm, and nm) and enamel properties of interest to analyze and compare mutant enamel with WT: mineral density, pigment, structure of prisms and crystals, cells, proteins, mechanical properties and elemental mapping. Within the macro scale (~1–10 mm) of enamel analysis **(A)** mineral density can be determined by enamel ashing. The representative 1-mm strips of mandibular incisor enamel dissected and processed for ashing are shown below the locations of stages of enamel development on mouse incisors in relation to molars (from Smith et al., 2011a). Note that ashing cannot be performed on highly mineralized and erupted enamel (after strip #5 or 6 mm from the apical loop). AL, apical loop; EM, start of enamel matrix formation; PS, presecretory stage; SEC, secretory stage; MAT, maturation stage; NTP, no Tomes' processes in ameloblasts; GM, gingival margin. **(B)** Pigment analysis of adult erupted WT incisor enamel using CIELAB color channels following high-resolution photography. Within the micro scale (~1–50 μm) of enamel analysis, there is a wide range of microscopy methods utilized to investigate enamel prism structure, ameloblasts and enamel proteins, including **(C)** light microscopy to analyze H&E stained paraffin sections of developing WT mouse molar enamel, and **(D)** SEM to analyze enamel prism structure. **(E)** μCT can be used to determine mineral density. Within the nano scale (50–100 nm), **(F)** TEM can be used to analyze enamel crystals, and additional high-resolution microscopy methods can be used to analyze the relationships between enamel prisms and the organic matrix (enamel proteins). Nanoindentation can be used to determine mechanical properties on a nano-scale, i.e., to measure differences between adjacent enamel prisms. Furthermore, elemental mapping of enamel can give information about the molecules that form enamel crystals **(G)**.

The most direct method to measure the mineral density of enamel is to perform the ashing technique, wherein adult rodent incisors are microdissected. Enamel is lifted off the dentin in 1-mm wide strips from secretory (apical) through maturation stages (incisal) with a scalpel blade (Figure 2A). The weight of each strip is measured before and after heating (ashing) to determine the relative mineral and organic content (Smith et al., 2005, 2009). The erupted portions of WT incisors are fully mineralized and thus too hard to cut with a scalpel blade and therefore not analyzed. Ashing has been used to determine mineral density in normal and genetically altered mice with mineralization defects: Enam^−/−^, Ambn^−^, Mmp20^−^, and KLK4^−/−^ (Smith et al., 2011b). While ashing is the most direct and accurate method to determine mineral density, it is destructive, time-consuming and lacks of reproducibility (Smith et al., 2011a; Schmitz et al., 2014). Furthermore, ashing requires advanced technical expertise to process the enamel strips.

Backscattered SEM (BSE) can also be used to quantitate mineralization of rodent enamel, either for visualization of enamel surface mineralization (Smith et al., 2009) or for investigating internal enamel mineralization in cross-section (Bronckers et al., 2013; Hu et al., 2014; Lyaruu et al., 2014). BSE relies on the linear relationship between the intensity of the BSE signal and the atomic number of a compound. Mineral densities are derived from the gray level of the BSE micrograph. Four-level color mapping has been used to aid visualization of mineral density differences across the thickness of rodent enamel (Smith et al., 2011b). For the most accurate assessment of mineral density, BSE should be used in combination with either μCT or ashing (Schmitz et al., 2014).

Micro-computed tomography (μCT) is a non-destructive method that utilizes differences in x-ray intensity before and after passing through an object (Figure 2E). Validity of enamel density measurements depends on calibration with hydroxyapatite (HA) standards (phantoms) (Schweizer et al., 2007), and the μCT has limitations in terms of the range of mineral densities it can detect. For example, μCT cannot be used to analyze secretory-stage enamel because of the similarities in densities between dentin and partially mineralized enamel. The lower range (secretory stage, hypoplastic, or hypomature enamel) of mineral densities are therefore not detectable. Ashing has been shown to be a more reliable method to analyze enamel with low mineral content (Schmitz et al., 2014). The upper range (over 1.2 g/cm^3^) of enamel mineralization must be extrapolated when using the Scanco μCT instrument, which is calibrated using HA phantoms between 0 and 1.2 g/cm^3^. μCT results correspond well to direct measurements by ashing and BSE (Schmitz et al., 2014).

μCT-based mineral density values of developing WT mouse incisor enamel range from 0.7 to 2.97 g HA/cm^3^, using phantoms with a broad range of densities (Schweizer et al., 2007) and the Skyscan μCT instrument. Fully mineralized WT incisor enamel measured using a Scanco μCT instrument was 2.7 g HA/cm^3^, a relative value (Bronckers et al., 2013; Lyaruu et al., 2014). It is important to note that interaction with the x-ray beam can change the sample properties (beam-hardening), which can greatly influence μCT measurements of mineral density (Burghardt et al., 2008; Fajardo et al., 2009; Hamba et al., 2012). Furthermore, manual contouring of volumes-of-interest (VOIs) for μCT can lead to inter- and intra-operator error. Therefore, enamel volume measurements are sensitive to user error and should be performed by a single operator (Schmitz et al., 2014). When reporting enamel mineral density values, it is important to understand the limitation of the method used and whether the reported values are relative or absolute.

### Enamel structure

Analyzing fully mineralized rodent enamel structure microscopically (other than SEM) is technically challenging due to the high level of mineralization and resulting tissue hardness. Sample preparation issues related to sectioning fully mineralized enamel or decalcification of enamel are well documented (Singhrao et al., 2010; Sun et al., 2012). To assess and compare enamel structure of mutant mice to WT, scanning electron microscopy (SEM) is commonly used. Rodent enamel is extracted, fixed in most cases, then fractured or sectioned before coating with gold and/or palladium for SEM analysis. Fracturing enamel allows visualization of enamel thickness and structure in fixed but otherwise unaltered tissue. Furthermore, fracturing of rodent incisors allows for visualization of enamel at specific developmental landmarks, which is useful to compare mutant enamel to WT. Fractured incisor enamel showed improvements in the rod structure of LRAP/Amelx^−/−^ mice, compared to Amelx^−/−^ incisor enamel which is aprismatic, indicative of partial rescue with the amelogenin splice variant (Gibson et al., 2009). When incisor enamel was fractured from mice overexpressing amelotin, the enamel was thin, with no decussating enamel prisms (Lacruz et al., 2012). Enam^+/−^ incisor enamel had decussating prisms but fractured differently than WT, while Enam^−/−^ incisor enamel was aprismatic (Hu et al., 2014).

To observe the enamel prism structure in more detail, plastic embedding allows for undecalcified samples to be sectioned. Furthermore, analysis of transverse sections through rodent incisors or molars by SEM allows for enamel thickness measurements. Ground sections are created by cutting thick sections (1–2 mm) of plastic embedded teeth and subsequent polishing of the section until the desired section thickness is reached. After the sections are polished, they are acid-etched, often with phosphoric acid to reveal the enamel rod organization by removing a thin layer of mineral from the polished surface (Figure 2D). Polished and etched first molar enamel from M180/LRAP/Amelx^−/−^ mice, when observed by SEM, showed that the enamel structure and thickness were similar to WT, and improved over M180/Amelx^−/−^ and LRAP/Amelx^−/−^, indicating complementarity of the transgenes M180 and LRAP in enamel formation (Gibson et al., 2011). Transmission electron microscopy (TEM) can be used to observe enamel crystallite morphology (Figure 2F) and requires ultrathin sections of 80–100 nm.

### Enamel proteins and ameloblasts

To identify enamel proteins in developing rodent enamel, immunogold labeling has been used. Colloidal gold particles are used to immunodetect to secondary antibodies which are in turn bound to primary antibodies designed to bind a specific protein. Gold is used for its high electron density which increases electron scatter to give high contrast dark spots under SEM or TEM. Mmp20 was localized to the forming outer enamel using electron immunogold staining (Bourd-Boittin et al., 2004). Dual-immunogold labeling has been used to visualize and quantify the presence of amelogenin and ameloblastin in secretory granules, and showed that amelogenin and ameloblastin are almost always packaged together for secretion, suggesting a functional relationship between these two enamel proteins (Zalzal et al., 2008). To understand the spatial localization of amelogenins in relation to the mineral crystallites, immunogold labeling with field emission SEM (FESEM) showed that amelogenins were located along the side faces of the apatite crystals (Du et al., 2009). TEM has also been used to visualize the secretion and localization of amelogenin in Enam^−/−^ mice (Hu et al., 2014).

To determine the distribution of the proteins ODAM and AMTN in maturation stage rodent incisors, postembedding colloidal gold immunocytochemistry showed localization to the basal lamina associated with maturation stage ameloblasts and suggested that the basal lamina is dynamic during the maturation process (Dos Santos Neves et al., 2012). The effects of overexpression of AMTN in cellular morphology of ameloblasts at different stages of enamel formation were observed by TEM (Lacruz et al., 2012).

Paraffin is most frequently used for histological embedding because it has a similar density as most soft tissues. However, mineralized tissues cannot be sectioned properly when embedded in paraffin because calcium and paraffin have different densities. After decalcification of enamel, the mineralized enamel layer is a blank space. Paraffin embedding is suitable for analysis of developing or embryonic enamel, since enamel mineralization takes place around birth, P0 in mice. The timing of mineralization should be considered when deciding which type of histological sections to use. In general, WT enamel tissue does not need decalcification if mice are 3 days postnatal or younger.

Ameloblasts can only be studied in sections of developing enamel (Figure 2C), as erupted enamel is acellular. Decalcified tissues can be cut more thinly than calcified tissue, allowing the sections to be studied under a wide range of magnification and microscopy techniques (TEM, environmental SEM, light microscopy, fluorescent microscopy, and confocal microscopy). However, it is extremely difficult to preserve mature enamel in decalcified sections. Decalcification removes the mineralized part of hard tissues, making the histologic examination of enamel less than optimal. Therefore, erupted enamel must be studied in calcified sections.

### Enamel mechanical properties

Mechanical property analysis to determine enamel functionality allows for the best endpoint assessment of enamel performance. Indentation is a non-destructive method to measure mechanical properties of materials and tissues. Generally, either microindentation hardness testing (microhardness) or nanoindentation has been used to determine enamel mechanical properties. Microhardness is measured using a diamond indenter with a specific geometry and makes an indent of about 50 μm into the tissue surface, using loads of up to 2N. The surface area of the indent is used to calculate the hardness value. Microhardness measurements of rodent enamel have suggested that its organic content significantly influences its mechanical properties (Baldassarri et al., 2008). Unfortunately microhardness testing is not optimal for measuring enamel from mice with genetically modified enamel genes, since these mutations often cause a hypoplastic (thin enamel) phenotype. While WT incisor enamel is approximately 100 μm thick, WT molar enamel is only about 50–60 μm thick (Gibson et al., 2011; Pugach et al., 2013). Amelogenin^−/−^ molar enamel is between 10 and 20 μm thick (Gibson et al., 2011; Pugach et al., 2013), which means that the microhardness indentation of 50 μm would extend beyond the KO enamel, making impossible to contain the indent with the enamel tissue. Furthermore, the loads used in microhardness testing may be too high to measure mutant rodent enamel. This problem was encountered when microhardness testing was attempted on incisor enamel from mice overexpressing the AMTN transgene, which was too brittle for measurements to be possible (Lacruz et al., 2012).

Nanoindentation uses small loads and tip sizes, with the indentation area measuring a few microns. The load and depth of penetration of the tip are plotted to create a load-displacement curve, which is used to calculate the mechanical properties: (1) Young's (elastic) modulus, or the elasticity of the material, and (2) hardness as a function of depth, which is slightly different from microhardness. Using a load of 1350 μN which produced an indentation of 3 μm, transgenic mouse enamel lacking the N-terminus of amelogenin (M180Δ A-FLAG) had 22% lower hardness and 24% lower elastic modulus than WT (Fong et al., 2003). Transgenic mouse enamel lacking the C-terminus of amelogenin (M180Δ B-FLAG) had 8% lower hardness and 18% lower elastic modulus. This decrease in mechanical properties was proposed to be due to defective amelogenin self-assembly due to misassembled nanospheres, compromising the integrity of the organic phase and thus the mechanical integrity. These data confirm the importance of the N- and C-termini of amelogenin in enamel formation (Fong et al., 2003).

**Figure 3:**
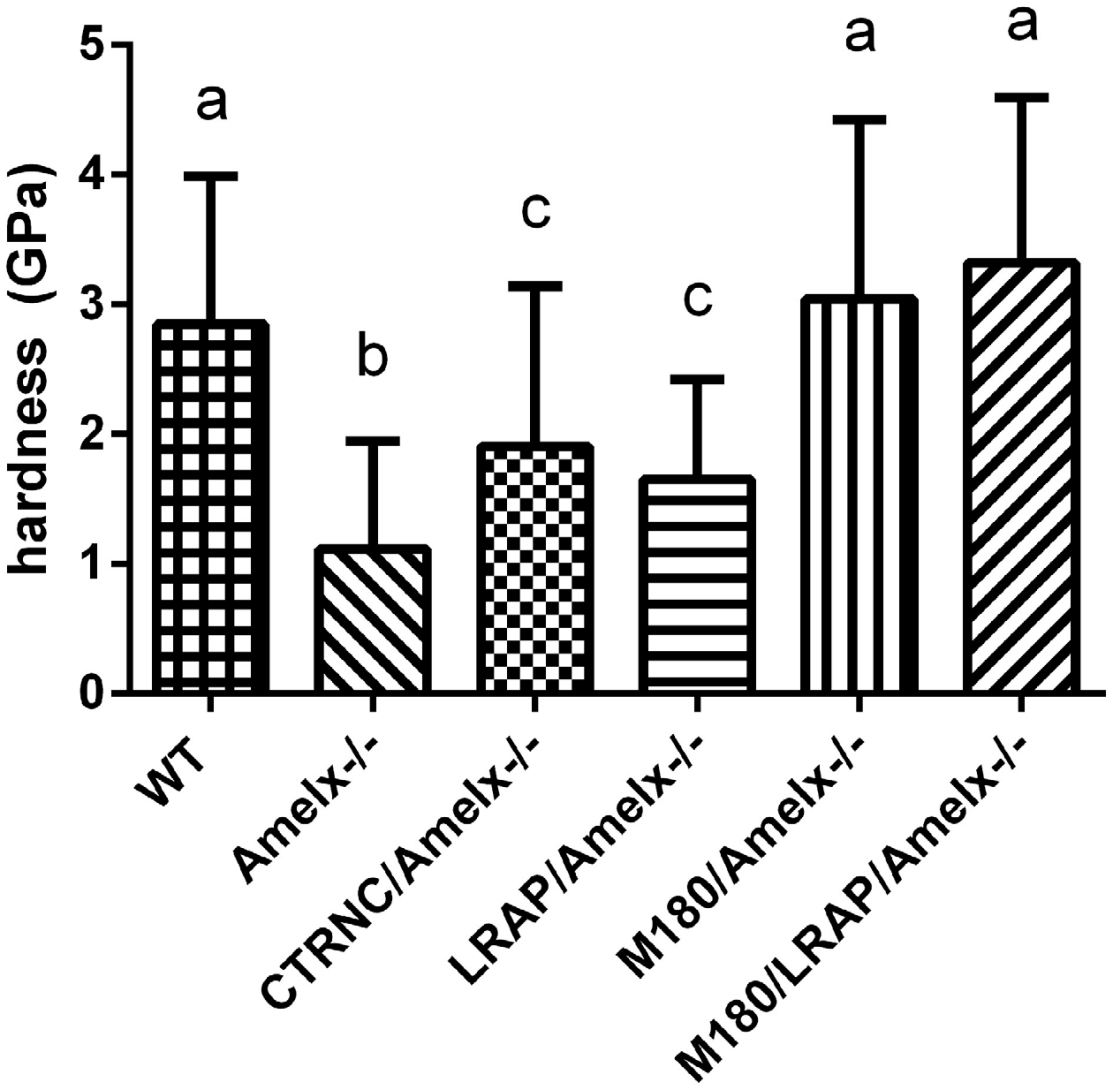
**Mouse molar enamel hardness measured by nanoindentation**. WT, M180/Amelx^-/-^, and M180/LRAP/Amelx^-/-^ (a) were not different from each other but were significantly harder than CTRNC/Amelx^-/-^ (c), LRAP/Amelx^-/-^ (c) and Amelx^-/-^ (b) enamel (*p* < 0.05). CTRNC/Amelx^-/-^ and LRAP/Amelx^-/-^ (c) were not different from each other but were significantly harder than Amelx^-/-^ enamel (*p* < 0.05).

Nanoindentation was used to measure mechanical properties of mice overexpressing different transgenes representing amelogenin cleavage products and isoforms in amelogenin null backgrounds. Amelogenin^−/−^ (KO) molar enamel has approximately 60% lower hardness and 58% lower elastic modulus than WT (Li et al., 2008; Pugach et al., 2013). Molar enamel from mice expressing only the most abundant amelogenin isoform (M180/Amelx^−/−^) had similar mechanical properties as WT, indicating rescue from this transgene even though the enamel thickness was not fully restored. Mice lacking the C-terminus of amelogenin (CTRNC/Amelx^−/−^) had 33% lower hardness and 38% lower elastic modulus than WT, again illustrating the importance of the C-terminus (Pugach et al., 2013). Recent nanoindentation data suggests that mice expressing the other abundant isoform, LRAP have molar enamel with 40% lower hardness and 34% lower elastic modulus than WT (Figure 3), an improvement over KO, which suggests that while the LRAP transgene was unable to rescue the mechanical properties as well as M180, it does play some role in the final mechanical properties of enamel. When the two transgenes, M180 and LRAP, are expressed in a KO background, the mechanical properties are unsurprisingly similar to WT (Figure 3).

### Enamel pigment

**Figure 4:**
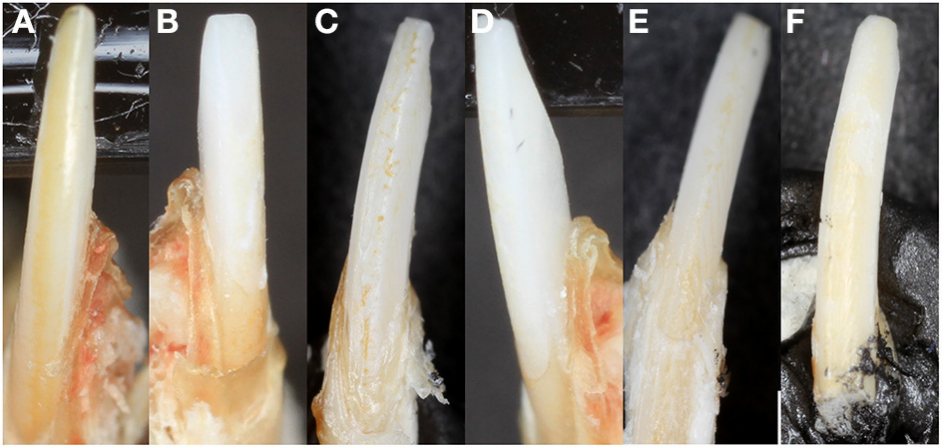
**Mouse incisor enamel pigment. (A)** WT enamel has a pigment due to iron content, **(B)** Amelx^-/-^ enamel is chalky white, **(C)** CTRNC/Amelx^-/-^ has some improvement in pigment from Amelx^-/-^, **(D)** LRAP/Amelx^-/-^ enamel is chalky white like Amelx^-/-^, **(E)** M180/Amelx^-/-^ enamel has some improvement in pigment from Amelx^-/-^, and **(F)** M180/LRAP/Amelx^-/-^ enamel has significantly more pigment than Amelx^-/-^ enamel.

Unlike human incisors, rodent incisors have a yellowish pigmentation due to a higher presence of iron in the outer enamel layer (Halse, 1972). It has been suggested that iron incorporation in enamel serves as a strengthening agent to resist cracking and abrasion, and that animals that feed on harder prey may have more iron (Motta, 1987). Many studies in which knockout or transgenic rodents are generated with alterations of enamel genes, loss of incisor pigment has been reported, indicating that iron incorporation is involved in normal enamel formation (Gibson et al., 2001; Paine et al., 2005) (Figure 2B). The variability of mouse incisor enamel pigment that is evident in Figure 4 is the result of mutations to different regions of a single gene, amelogenin, and its isoforms. A recent study confirmed that iron is present in rodent molars in addition to their incisors, indicating the iron is an essential component for rodent enamel formation (Wen and Paine, 2013). It was therefore suggested that iron incorporation may be final refinement for enamel mineralization, providing extra strength and acid resistance. However, the presence of iron is linked to erupting nature of the rodent tooth since there is a significantly higher iron accumulation in incisors compared to molars (Wen and Paine, 2013).

Morphometric measurements of rodent incisor color and whiteness are essential to aid in interpretation of phenotype outcomes caused by genetic alterations of enamel genes. A detailed method to measure rodent incisor color and whiteness has been recommended (Coxon et al., 2012). Incisors are held in black modeling clay and imaged in 2D with an SLR camera using an established image analysis system (Brook et al., 2007) and macro photo lens. Using Adobe Photoshop, red, green, and blue (RGB) outputs were converted to CIELAB color space and whiteness using previously described methods (Guan et al., 2005; Smith et al., 2008).

Quantification of mutant enamel color and whiteness phenotypes using established methods is recommended for aiding in the understanding of the role of enamel proteins in its formation, and can allow for comparisons of mutant rodent models between investigators. The CIELAB or CIE (Kuehni, 1976) L^*^a^*^b values are perceptually based: L^*^ is lightness related to physical intensity of a color, a^*^ represents the red-green axis, and b^*^ represents the yellow-blue color axis. The CIE whiteness index (WIC) has been used to describe tooth color for making porcelain veneers and whitening, where white is L^*^ = 100, a^*^ = 0 and b^*^ = 0. Mutant mouse incisor enamel has been measured using this technique and *Amelx* and *Enam* mutant mice had significantly lower yellow/blue (b) values and higher lightness (L) and whiteness (WIC) values than their WT counterparts (Coxon et al., 2012).

Mouse models with genetically modified enamel genes have been generated for the past two decades using a variety of methods including transgenics, knockouts, conditional knockouts, and knockins. These valuable research tools continue to be the most direct way to study enamel formation *in vivo* and Amelogenesis Imperfecta. Investigators have utilized a variety of methods to determine the outcome of generating mutant enamel, to observe mineral content, structure, mechanical properties, pigment, and in the case of developing enamel, ameloblasts (Figure 2G). In order for data characterizing enamel phenotypes from mice with mutated enamel genes to be compared to each other, it is necessary to have standardized workflow of analytical methods. Enamel development and mineralization is a dynamic and complex process, and correlating similarly measured data between mouse models could allow for increased understanding of the process of forming this extraordinarily intricate enamel tissue.

### Conflict of interest statement

The authors declare that the research was conducted in the absence of any commercial or financial relationships that could be construed as a potential conflict of interest.
